# Role of ER Stress Response in Photodynamic Therapy: ROS Generated in Different Subcellular Compartments Trigger Diverse Cell Death Pathways

**DOI:** 10.1371/journal.pone.0032972

**Published:** 2012-03-05

**Authors:** Irena Moserova, Jarmila Kralova

**Affiliations:** Department of Molecular Virology, Institute of Molecular Genetics, Academy of Sciences of the Czech Republic, Prague, Czech Republic; Karolinska Institutet, Sweden

## Abstract

We have analyzed the molecular mechanisms of photoinduced cell death using porphyrins with similar structure differing only in the position of the ethylene glycol (EG) chain on the phenyl ring. Meta- and para-positioned EG chains targeted porphyrins to different subcellular compartments. After photoactivation, both types of derivatives induced death of tumor cells via reactive oxygen species (ROS). Para derivatives pTPP(EG)4 and pTPPF(EG)4 primarily accumulated in lysosomes activated the p38 MAP kinase cascade, which in turn induced the mitochondrial apoptotic pathway. In contrast, meta porphyrin derivative mTPP(EG)4 localized in the endoplasmic reticulum (ER) induced dramatic changes in Ca^2+^ homeostasis manifested by Ca^2+^ rise in the cytoplasm, activation of calpains and stress caspase-12 or caspase-4. ER stress developed into unfolded protein response. Immediately after irradiation the PERK pathway was activated through phosphorylation of PERK, eIF2α and induction of transcription factors ATF4 and CHOP, which regulate stress response genes. PERK knockdown and PERK deficiency protected cells against mTPP(EG)4-mediated apoptosis, confirming the causative role of the PERK pathway.

## Introduction

Photodynamic therapy (PDT) of cancer is based on tumor-specific accumulation of a photosensitizer, followed by irradiation with visible light, resulting in cell death and tumor ablation [1]. Activated photosensitizers transfer energy to molecular oxygen, generating reactive oxygen species (ROS) [2]. ROS formed upon irradiation, namely singlet oxygen, have a limited lifetime and ability to migrate from the site(s) of their formation. Thus, they interact with biologic substrates in the sites of photosensitizer localization. The molecular nature of the photo-oxidized targets has profound influence on the signaling pathways and mode of cell death initiated by PDT. Effective photosensitizers localize in the mitochondria, endoplasmic reticulum (ER), Golgi apparatus, lysosomes and plasma membrane, or they may have multiple targets [3]. It must be mentioned that PDT-mediated apoptotic/necrotic outcomes are influenced by the cell type, identity and concentration of the photosensitizer, and light doses used in the PDT protocol. The type of cell death switches from apoptosis to necrosis with the increasing intensity of the insult.

There are extensive reports on the central role of mitochondria in the apoptotic cell death, where intrinsic and extrinsic cell death stimuli activating different but often overlapping signaling pathways converge to induce permeabilization of the mitochondrial membranes (MMP). MMP results in mitochondrial depolarization and release of several apoptogenic proteins into the cytosol, including activators of caspase-9 such as cytochrome c, through the formation of a heptameric complex called the apoptosome [reviewed in [3], [4]].

The evidence was collected showing an important role of the endoplasmic reticulum (ER) in the maintenance of intracellular calcium homeostasis, protein synthesis, posttranslational modification, and proper folding of proteins as well as their sorting and trafficking. Many stimuli can cause stress resulting in apoptosis [5] through the unfolded protein response (UPR) and Ca^2+^ signaling mechanisms, which together may activate calpain and cleavage of caspases-4/12 [5], [6], [7], [8].

Generally, ER stress is sensed by three integral stress receptors, pancreatic ER kinase (PKR)-like ER kinase (PERK), activating transcription factor 6 (ATF6), and inositol requiring enzyme 1 (IRE1) [9]. These ER transmembrane proteins are kept in inactive state through direct association of their luminal domain with the ER chaperone Bip/GRP78 (glucose-regulated protein). However, upon accumulation of unfolded proteins, GRP78 dissociates from these molecules, which leads to their activation. PERK phosphorylates the α subunit of eukaryotic initiation factor 2 (eIF2α) to promote translation of activating transcription factor 4 (ATF4) [10], which subsequently upregulates the expression of the proapoptotic protein CHOP (C/EBP-homologous protein-10 also known as GADD153) [11].

The antiapoptotic Bcl-2 protein was recognized as a molecular target for several mitochondria- and ER-localized photosensitizers, and its photodamage leads to the release of cytochrome c from mitochondria, activation of caspase-3 and initiation of the apoptotic program [12], [13], [14], [15], [16], [17]. An immediate loss of the sarco/endoplasmic reticulum Ca^2+^-ATPase-2 (SERCA2) protein levels causing disruption of Ca^2+^ homeostasis and cell death was described for ER-associated hypericin [18]. In summary, PDT may bring along the calcium release from ER, inhibition of Bcl-2 and other Bcl-2 family members located in the ER or mitochondrial membranes, direct mitochondrial photodamage, lysosomal fragmentation, or relocalization of the photosensitizer. This suggests that photodamage can rapidly spread from the primary site to other subcellular locations [19], [20], [21].

The so far most extensively studied photosensitizers are porphyrins. In our previous study, we reported that ethylene glycol (EG)-porphyrin derivatives represent a unique model enabling study of the structure-activity relationship in the context of identical molecules differing only in the glycol chain being in either the meta-phenyl or the para-phenyl position [22]. This subtle change affected uptake, intracellular localization and PDT efficacy. Porphyrin with EG chain in para position (compound **4**) was localized mainly in the lysosomes, whereas porphyrin with EG chain in meta position (compound **2**) was mostly found in the ER. Moreover, the derivative with EG chain in meta position displayed superior PDT efficacy, leading to permanent ablation of human mammary carcinoma (MDA-MB-231) in immunodeficient mice [22]. Extended analysis of the fluorinated analog carrying the EG chain in para position (PORF-TEG) revealed that although primarily accumulated in lysosomes, it induces mitochondria-driven apoptosis and operates mainly via activation of the p38 signaling cascade [23].

The present study was undertaken to further explore the commitment events and modality of cell death by comparing the signaling pathways induced by meta and para EG-porphyrin photoactivation in two tumor cell lines of different origin. Here, we demonstrate that EG-porphyrin-mediated PDT induces ER stress, involving calcium homeostasis disruption, and activation of the PERK pathway and caspase-4/12. Moreover, we provide evidence that ER stress plays a causative role in PDT mediated by the meta derivative, but only a marginal role in PDT mediated by the para derivative. Finally, our study brings better understanding of the role of diverse triggering pathways in the process of cell death following PDT.

## Results

**Figure 1:**
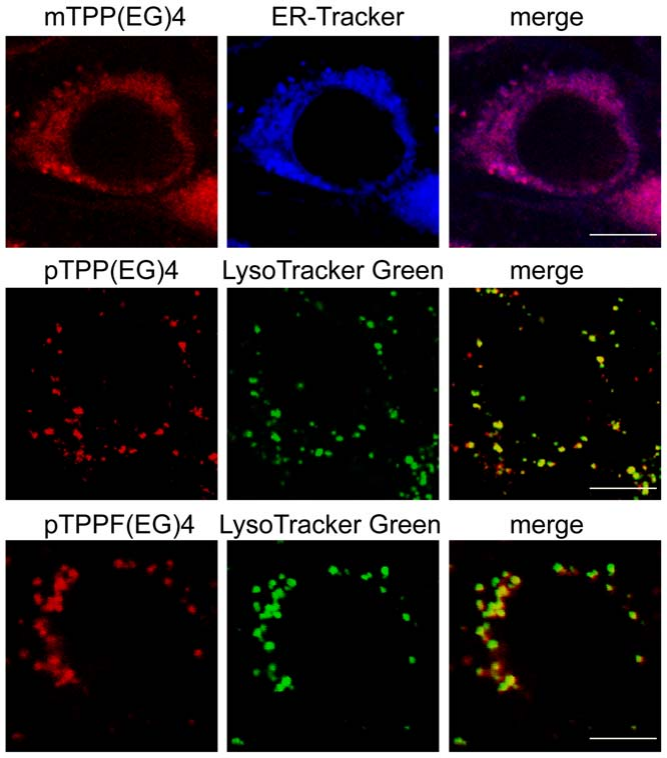
Subcellular localization of EG-porphyrin derivatives. 4T1 cells were loaded with EG-porphyrin derivatives (left panels) and specific probes for endoplasmic reticulum (ER) and lysosomes (middle panels). An overlay of EG-porphyrin derivatives fluorescence with ER-Tracker or LysoTracker fluorescence is shown in the right panels. Scale bars: 10 µm.

EG-porphyrin derivatives implemented in our study displayed different intracellular localization depending on the position of the EG chain. Meta position directed preferential accumulation in the endoplasmic reticulum [mTPP(EG)4)], while para position [pTPP(EG)4 and pTPPF(EG)4] led to the localization in lysosome-like structures (Fig. 1), as determined by co-staining with organelle-specific probes LysoTracker and ER-Tracker.

### MAP kinases (MAPK) and their role in EG-porphyrin-mediated apoptosis

**Figure 2:**
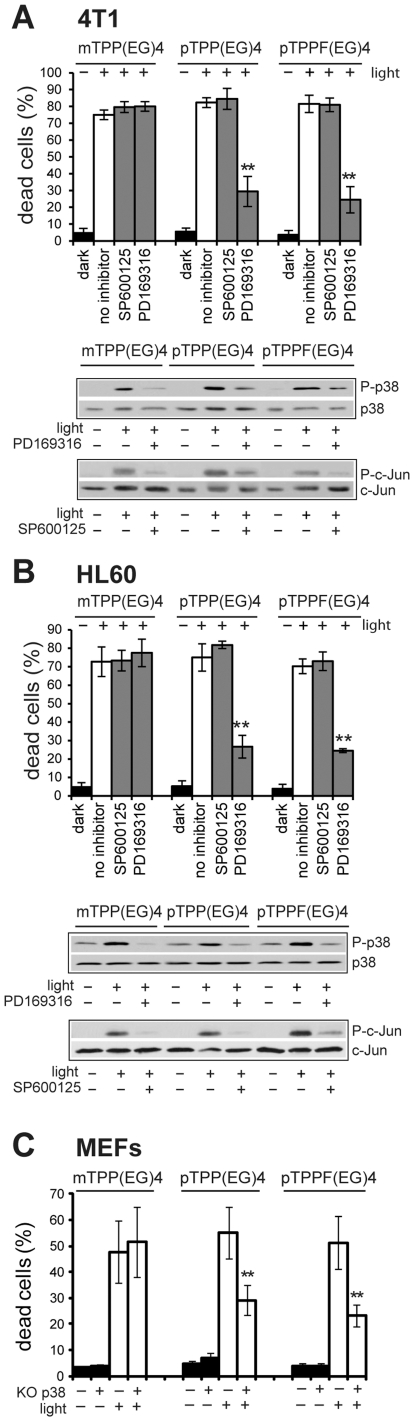
Effect of kinase inhibitors on EG-porphyrin-mediated phototoxicity in 4T1 and HL60 cells. 4T1 cells (**A**) or HL60 cells (**B**) were loaded with EG-porphyrin derivatives for 16 h, washed and then preincubated for 1 h JNKII inhibitor (5 µM) and PD169316 (10–20 µM) before light exposure. After irradiation the cells were further cultivated and cell viability was determined 24 h post-PDT by the trypan blue exclusion method. Cells treated with EG-porphyrin derivatives without irradiation were used as controls (dark). The percentage of dead cells was expressed as the mean ± SD (n = 4). ** P<0.01 represents statistical differences between PDT-treated cells vs. PDT-treated cells in the presence of JNKII or PD169316. For Western blot analysis cells were pre-incubated for 1 h with JNKII inhibitor (SP600125) or p38 inhibitor (PD169316) and 2 h post-irradiation analyzed with specific antibodies recognizing the phosphorylated forms of p38 and c-Jun by Western blot (P-p38 and P-c-Jun, respectively). Equal loading is demonstrated by total c-Jun and p38 protein. (**C**) Effect of p38 deletion on apoptotic cell death. Mouse embryo fibroblasts (MEFs) with intact (wt) and inactivated *p38*α−/− (KO) gene were exposed to EG-porphyrin-mediated PDT. Cell viability was evaluated 24 h post-PDT by the trypan blue exclusion method. **P<0.01 represents statistical differences between wt MEF vs. KO MEF exposed to PDT.

It is known that intracellular localization of photosensitizers strongly affects the mechanism of cell death. Our previous study revealed an important role of p38 MAPK signaling in the cell death induced by fluorinated derivative PORF-TEG [23], designated in the current study as pTPPF(EG)4. Similar p38 and JNK activation patterns were observed using mTPP(EG)4 = compound **2**, pTPP(EG)4 = compound **4** [22]. The next step was to examine whether MAPK activation is required for the apoptotic process mediated by mTPP(EG)4 and pTPP(EG)4 as well. For this purpose, we used pharmacological JNK kinase inhibitor (SP600125) and p38-specific inhibitor (PD169316). For comparison we included the previously studied pTPPF(EG)4. The inhibition of JNK and p38 activity by these compounds in HL60 and 4T1 cells was verified by Western blot analysis (Fig. 2A and 2B -lower panels). Importantly, the inhibition of JNK kinase did not influence EG-porphyrin-mediated cell death, thereby excluding JNK involvement in this process. On the other hand, the inhibition of p38 MAPK (Fig. 2A and 2B) or knockout of the *p38*α gene (Fig. 2C) resulted in reduction of the apoptotic cell death induced by both para derivatives [p-TPP(EG)4 and p-TPPF(EG)4 (Fig. 2A and 2B - upper panels)]. No effect was observed in the case of meta derivative mTPP(EG)4. Thus, p38 MAPK activation seems to play a substantial role in the induction of apoptosis for both pTPP(EG)4 and pTPPF(EG)4 derivatives localized to lysosomes, but not for ER-localized mTPP(EG)4.

### ROS as primary triggers of cell death signals in EG-porphyrin-mediated cell death

**Figure 3:**
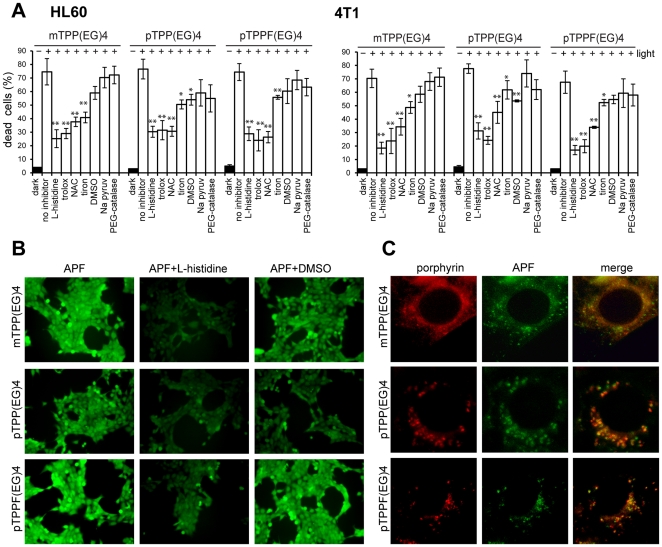
Role of ROS generation in EG-porphyrin-mediated cell death. (**A**) Effect ROS scavengers on cell viability after PDT. 4T1 cells were loaded with EG-porphyrin derivatives for 16 h, washed and then pre-incubated for 1 h with L-histidine (20 mM), trolox (5 mM), NAC (15 mM), tiron (10 mM), DMSO (1%), PEG-catalase (300 U/ml) and sodium pyruvate (Na Pyruv) (10 mM) before light exposure. Similarly, HL60 cells were pre-incubated with L-histidine (20 mM), trolox (4 mM), NAC (5 mM), tiron (10 mM), DMSO (1%), PEG-catalase (300 U/ml) and sodium pyruvate (Na Pyruv) (10 mM). After irradiation the cells were further cultivated and cell viability was determined 24 h post-PDT by the trypan blue exclusion method. Cells treated with EG-porphyrin derivatives without irradiation were used as controls (dark). The percentage of dead cells was expressed as the mean ± SD (n = 3). *P<0.050, ** P<0.01 represents statistical differences between PDT-treated cells vs. PDT-treated cells in the presence of L-histidine, trolox, NAC, tiron and DMSO. (**B**) ROS detection. 4T1 cells incubated with EG-porphyrin derivatives were washed and loaded with 10 µM APF(3′-(p-aminophenyl) fluorescein) in the presence or absence of L-histidine (20 mM) or DMSO (1%) for 30 min. Dye-loaded cells were then stimulated with UV light for 10 s under a DM IRB microscope (Leica) and fluorescence images were acquired immediately using a ×10 objective. (**C**) *In situ* detection of ROS generation. 4T1 cells grown on coverslips were sequentially loaded with porphyrin derivatives and APF probe. After 30 min incubation with the APF probe cells were exposed to UV light for 10 s and fluorescence images were acquired immediately using a ×63 objective. Fluorescent images of EG-porphyrin derivatives (left panels), APF probe (middle panels) and their overlay (right panels). Scale bars: 10 µm.

In numerous PDT studies it has been demonstrated that generation of ROS is responsible for the initiation of cell death. To analyze the contribution of particular oxygen species we implemented in our study various scavengers and antioxidants and followed their impact on EG-porphyrin-mediated cell death. HL60 and 4T1 cells loaded with EG-porphyrins were pre-incubated for 1 h with singlet oxygen and ROS scavengers and were then exposed to the light. The cell viability evaluated next day was dependent on the used porphyrin derivative, scavenger, and partly on the cell type. Generally, in both cell types the most dramatic reduction of cell death was caused by singlet oxygen (^1^O_2_) quencher L-histidine, by trolox and by NAC, known as radical scavengers and antioxidants (Fig. 3A). Tiron, claimed to be a superoxide anion (^•^O_2_^−^) scavenger, was effective mainly in the context of mTPP(EG)4 in HL60 cells, and DMSO as hydroxyl radical (^•^OH) scavenger was moderately effective and reached a significant level only in the case of pTPP(EG)4. PEG-catalase and sodium pyruvate as hydrogen peroxide (H_2_O_2_) scavengers slightly attenuated demise of cells treated with para derivatives but not with meta derivative. To dissect the role of ROS species triggering apoptotic cell death, we used a 3′-p-(aminophenyl) fluorescein (APF) probe, which was successfully used to monitor the formation of both ^1^O_2_ and ^•^OH in L1210 cells subjected to benzoporphyrin-mediated PDT according to Price et al. [24]. APF, unlike 2′,7′-dichlorodihydrofluorescein, was found resistant to light-induced autoxidation, and it is therefore more appropriate for monitoring formation of both ROS in the context of PDT [25]. Cells loaded with EG-porphyrins were incubated with the APF probe as described in Materials and Methods in the presence or absence of L-histidine or DMSO. Immediately after incubation the cells were exposed to UV light for 10 s and pictures of APF fluorescence activated by ROS were collected by a camera under identical settings using a ×10 objective (Fig. 3B). Control cells without porphyrins, when incubated with the APF probe after UV exposure, did not show any fluorescence (data not shown). L-histidine treatment dramatically reduced APF fluorescence, and consistently with phototoxicity data reflects the major role of ^1^O_2_ formation in all three EG porphyrin derivatives. According to Price [24], the portion of the fluorescence quenched by DMSO represents the contribution of ^•^OH. The significant reduction of toxicity and APF fluorescence in the case of pTPP(EG)4 photooxidation therefore indicated important participation of ^•^OH.

The photogenerated singlet oxygen has a short lifetime and a limited diffusion path in biological systems, indicating that primary molecular targets of the photodynamic process must reside within a few nanometers of the photosensitizer. As shown in Fig. 1, para derivatives preferentially accumulate in the lysosomes and the meta derivative localizes in ER; hence the immediate generation of ROS should occur in the lysosomes and ER, respectively. We therefore used the APF probe to monitor the ROS generation induced by exposure to UV light as described above. APF and porphyrin fluorescence was visualized under a fluorescence microscope using a ×63 objective. As shown in Fig. 3C, APF fluorescence was generated rapidly within seconds after UV light exposure and mostly overlapped with porphyrin location. Thus, the immediate ROS generation corresponds to primary porphyrin compartmentalization.

### Photoactivation of EG-porphyrin derivatives induces various levels of ER stress implementing Ca^2+^

**Figure 4:**
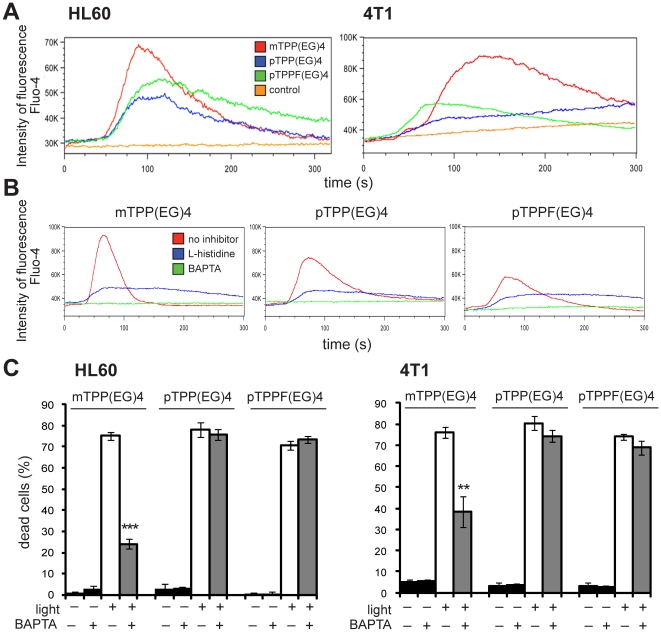
The role of Ca^2+^ in photoinduced apoptosis by EG-porphyrin derivatives. (**A**) Intracellular calcium levels in HL60 and 4T1 cells. The effect EG-porphyrin derivatives on cytosolic Ca^2+^ levels was monitored by flow cytometry of laser-illuminated cells containing fluorescent Ca^2+^ indicator Fluo-4-AM (4 µM) (Molecular Probes). Cells treated in the same way but without porphyrin were used as controls. (**B**) Effect of BAPTA and L-histidine on the intracellular Ca^2+^ level. HL60 cells were loaded with EG-porphyrin derivatives for 16 h, washed and then preincubated for 1 h with ROS scavenger L-histidine (20 mM) or 2 h with calcium chelator BAPTA-AM (5 µM). The cytosolic Ca^2+^ level was monitored by flow cytometry using fluorescent Ca^2+^ indicator Fluo-4-AM (4 µM). (**C**) Effect of BAPTA-AM preincubation on photoinduced apoptosis. Cells loaded with EG-porphyrin derivatives were pre-incubated with the membrane-permeable intracellular Ca^2+^ chelator BAPTA-AM (5 µM HL60, 10 µM 4T1) for 2 h to inactivate released Ca^2+^ and then irradiated with 2.5 Jcm^−2^ of light at 500±20 nm. This corresponds to an LD_70_ PDT dose. Cell viability was determined 24 h post-photodynamic therapy by the trypan blue exclusion method. The percentage of dead cells was expressed as mean ± SD (n = 4). **P<0.01, ***P<0.001 represents statistical differences between PDT-treated cells vs. PDT-treated cells in the presence of BAPTA-AM.

mTPP(EG)4 photoinduced apoptosis seems to be ruled by some other mechanism(s) than that induced by the para derivatives. In order to find out the responsible signaling pathways we focused on ER, the main store of Ca^2+^, where mTPP(EG)4 is prevalently localized. The impact of changes in Ca^2+^ homeostasis on the cell death was evaluated. Firstly, cytosolic Ca^2+^ levels in cells loaded with EG-porphyrin derivatives were monitored by flow cytometry using fluorescent Ca^2+^ indicator Fluo-4-AM. A major rise of (Ca^2+^)cyt was observed within one minute after the cells were exposed to the laser beam. A similar elevation was induced in 4T1 cells by employing thapsigargin (TG), a specific mobilizer of the ER-Ca^2+^ store, and ionomycin, a widely known ionophore, which releases calcium from all cellular calcium stores (Fig. S1). Our experiments indicate that the cytosolic levels of Ca^2+^ are increased in the presence mTPP(EG)4 and to a lower extent in the presence of pTPP(EG)4 or pTPPF(EG)4 (Fig. 4A).

When the cells were pre-incubated with a calcium-inhibiting agent, BAPTA-AM (Molecular Probes), which diminishes intracellular Ca^2+^ concentration, Fluo-4 signal was completely abrogated in HL60 cells loaded with all three porphyrin derivatives (Fig. 4B). A similar inhibitory effect was observed when the cells were pre-treated with ^1^O_2_ scavenger L-histidine (Fig. 4B). This observation proves that ROS generation is a primary event leading to a (Ca^2+^)cyt rise. Moreover, cells pre-loaded with membrane-permeable intracellular Ca^2+^ chelator BAPTA-AM exhibited a significantly reduced level of mTPP(EG)4-mediated apoptosis (Fig. 4C). Thus, the phototoxicity of mTPP(EG)4 is at least partly dependent on the rise of (Ca^2+^)cyt in 4T1 and HL60 cells, but other cell death mechanism(s) not mediated by Ca^2+^ may also be implicated. In contrast, pTPP(EG)4 and pTPPF(EG)4-induced cell death was not affected by BAPTA-AM preincubation. It indicates that ER stress demonstrated by a moderate (Ca^2+^)cyt elevation in para derivatives does not play a critical role in the initiation of apoptosis. Cell death is mediated by another mechanism allowing full development of apoptosis in the presence of BAPTA. Furthermore, we were not able to detect any contribution of extracellular Ca^2+^ influxes on the phototoxicity of either meta or para porphyrin derivatives using calcium-free medium or pre-treatment with extracellular chelator EDTA (data not shown).

**Figure 5:**
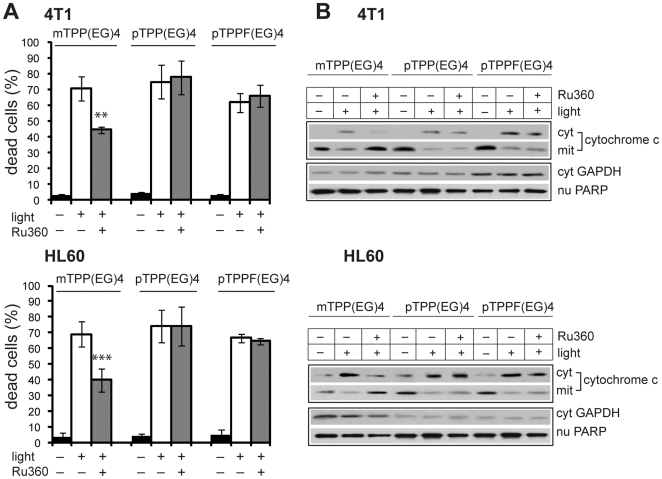
Effect of Ru360. (**A**) Cells loaded with EG-porphyrin derivatives were pre-treated with Ru360 (5 µM 4T1, 4 µM HL60) for 1 h to inhibit Ca^2+^ mitochondrial uptake. After irradiation the cells were further incubated in medium and their viability was determined 24 h later by the trypan blue exclusion method. The percentage of dead cells was expressed as the mean ± SD (n = 3–6). **P<0.01, ***P<0.001 represents statistical differences between PDT-treated cells vs. PDT-treated cells in the presence of Ru360. (**B**) Cells were preincubated for 1 h with Ru360 (4–5 µM) and 2 h post-irradiation the cytosolic, mitochondrial and nuclear extracts were prepared and analyzed by Western blotting with specific antibody recognizing cytochrome c, GAPDH and PARP. Results are representatives of at least three experiments.

Efflux of Ca^2+^ from the stressed ER and uptake of the released Ca^2+^ by mitochondria have been reported to activate/potentiate the intrinsic apoptotic pathway [26]. Therefore, we examined the effect of blocking mitochondrial Ca^2+^ uptake by Ru360, a specific uniporter inhibitor of the mitochondrial Ca^2+^. Data collected from these experiments displayed a reduced level of mTPP(EG)4-mediated phototoxicity (Fig. 5A). Moreover, the cytosolic rise of cytochrome c induced by EG-porphyrin photoactivation was prevented by Ru360 pretreatment in mTPP(EG)4 but not in pTPP(EG)4- or pTPPF(EG)4-loaded cells (Fig. 5B). Correspondingly, cytosolic reduction of the cytochrome c level was accompanied by its higher content in mitochondrial extract. In summary, our findings demonstrate that mTPP(EG)4-mediated apoptosis is inhibited either by calcium chelator BAPTA-AM or by mitochondrial uniporter inhibitor Ru360. Hence, we speculate that the flux of ER-derived Ca^2+^ into the mitochondria is likely involved in the initiation and development of apoptosis in the mTPP(EG)4 PDT model.

**Figure 6:**
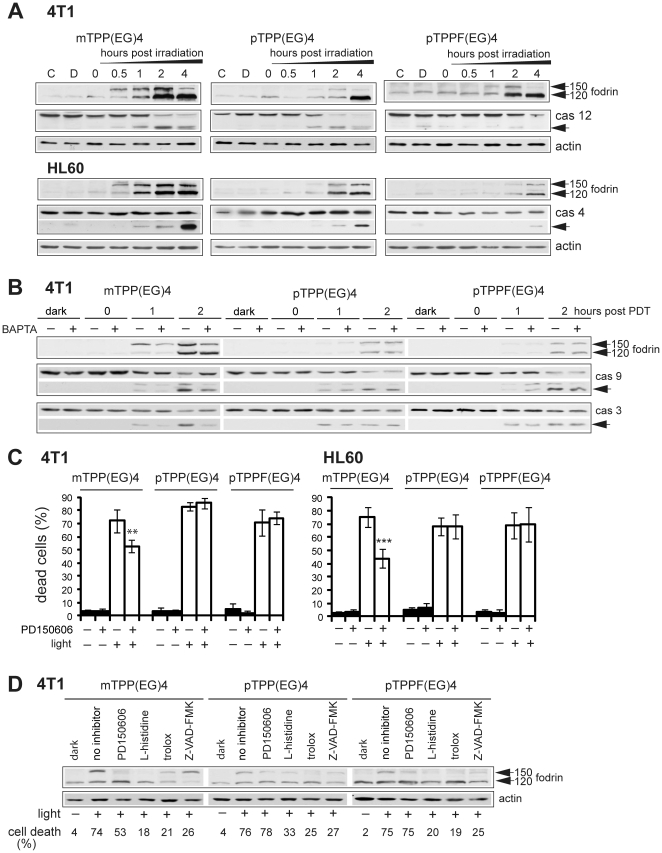
Effect of EG-porphyrin derivatives on Ca^2+^ signaling pathway. (**A**) Western blot analysis of fodrin, caspase-12 (4T1 cells) and caspase-4 (HL60 cells). Cells treated with EG-porphyrin derivatives were harvested at various times after irradiation and subjected to Western blot analysis with antibodies recognizing fodrin and caspase-12. Reprobing with β-actin antibody was used to confirm equal loading. The arrowheads point to activated forms. (**B**) Pretreatment of 4T1 cells with BAPTA-AM (10 µM) resulted in the inhibition of fodrin, caspase-9 and caspase-3 activation caused by mTPP(EG)4-PDT. 4T1 cells loaded with porphyrin derivatives were pre-incubated for 2 h with BAPTA-AM and then exposed to light (2.5 Jcm^−2^). At various times after irradiation the cells were lysed and analyzed with antibody recognizing fodrin, full-length and cleaved p39 form of caspase-9 and full-length and cleaved p17 form of caspase-3 on Western blots. (**C**) Effect of calpain inhibitor PD150606 on viability of 4T1 and HL60 cells. Cells were incubated with PD150606 (20 µM) for 1 h and then irradiated. Cell viability was estimated after 24 h by the trypan blue exclusion method. The percentage of dead cells was expressed as the mean ± SD (n = 4–5). **P<0.01, ***P<0.001 represents statistical differences between PDT-treated cells vs. PDT-treated cells in the presence of PD150606. (**D**) The effect of calpain inhibitor, ROS scavengers (L-histidine and trolox), and caspase inhibitor (Z-VAD-FMK) on the activation of fodrin. Cells were treated as described in Materials and Methods, harvested 1 h post PDT treatment and analyzed by Western blot. Equal protein loading is demonstrated by actin reprobing. The viability of cells subjected to simultaneous treatment done in parallel is presented under the Western panel.

Next, we determined the effect of EG-porphyrins on the activation of calpain and relevant caspases. The activation of calpain was monitored by cleavage of fodrin (Fig. 6A), a marker of calpain action [27]. Fodrin is known to be cleaved by both calpains and caspase-3, resulting in a calpain-specific 150 kDa fragment and a 120 kDa fragment generated by caspase-3. In 4T1 cells subjected to mTPP(EG)4-PDT, fodrin cleavage appeared 0.5 h post irradiation and correlated with caspase-12 activation (Fig. 6A, top panels). In contrast, lower activation with delayed kinetics of fodrin and caspase-12 was determined in cultures treated with para derivatives. Similar results were obtained in human cells (HL60) when tested for fodrin and caspase-4, human homolog of caspase-12 (Fig. 6A, lower panels). In addition, BAPTA-AM pretreatment attenuated meta porphyrin-induced activation of fodrin, caspase-9 and caspase-3 but did not affect that of para derivatives (Fig. 6B).

Further, we tested the influence of calpain inhibitor PD150606 on the viability of 4T1 and HL60 cells exposed to PDT. Pretreatment with calpain inhibitor reduced mTPP(EG)4-mediated phototoxicity, thereby bringing an additional proof for the engagement of the Ca^2+^ pathway in the mechanism of cell death (Fig. 6C). Calpain inhibitor, however, had no impact on cell death induced by para derivatives. To determine the cause, we followed the effect of calpain inhibitor, ROS scavengers and caspase inhibitor on the fodrin cleavage. Cells subjected to treatment with the inhibitors were harvested for protein analysis and in a parallel experiment was cell viability evaluated next day (Fig. 6D). ROS inhibitors (L-histidine, trolox) suppressed fodrin cleavage and phototoxicity of all three photosensitizers. In contrast, calpain inhibitor reduced fodrin cleavage in all of them but attenuated cell death of the meta derivative only, by 21%. The caspase inhibitor (Z-VAD-FMK) reduced the level of caspase-specific 120 kDa fodrin fragment and attenuated PDT-induced cell death mediated by both meta and para derivatives (Fig. 6D). Beside that, after treatment with para derivatives, a “calpain-specific” 150 kDa fragment appeared in weakened form. This might indicate participation of caspases in the activation of the “calpain-specific” 150 kDa fodrin fragment.

**Figure 7:**
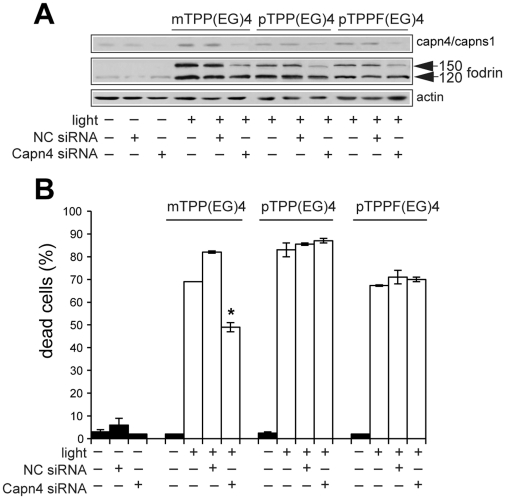
Effect of Capn4 siRNA on the demise of PDT-treated cells. (**A**) Western blot analysis of capn4/capns1 and fodrin in 4T1 cells analyzed on the 3^rd^ day after Capn4 siRNA transfection. Transfected cells were subjected to EG-porphyrin-mediated PDT, harvested and resolved by Western blot analysis. Equal protein loading is demonstrated by actin reprobing. (**B**) Viability of cells transfected with Capn4 siRNA. Transfected and control cells were subjected to EG-porphyrin-mediated PDT and cell viability was monitored 24 h later. The percentage of dead cells was expressed as the mean ± SD (n = 3). *P<0.05 represents the statistical difference between transfected PDT-treated cells vs. non-transfected or control siRNA-transfected PDT-treated cells.

To confirm the importance of the calpain activation in EG porphyrin-mediated phototoxicity, we knocked down the expression of Capn4 using specific siRNA. Transfection of Capn4 siRNA, but not control siRNA (NC), decreased the level of endogenous Capn4/Capns1 protein and reduced fodrin cleavage (Fig. 7A). Moreover, Capn4 siRNA significantly reduced the amount of dead cells in mTPP(EG)4-treated cells (Fig. 7B). Despite that knockdown of Capn4 decreased fodrin cleavage also in para derivatives, no effect on cell viability was observed (Fig. 7B). In summary, all these experiments confirm an important role of calpain activation in mTPP(EG)4 but not pTPP(EG)4 and pTPPF(EG)4-induced cell death.

### ER-localized mTPP(EG)4 causes severe ER stress and subsequent activation of several UPR target genes

**Figure 8:**
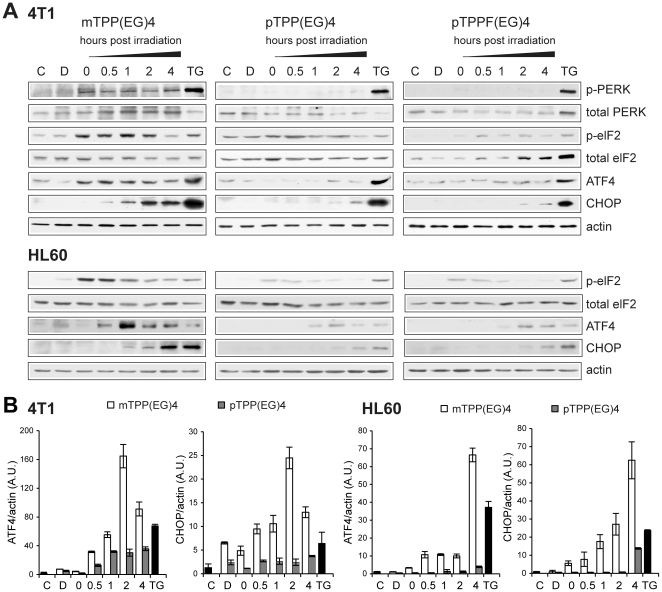
Kinetics of PERK pathway activation by EG-porphyrin-mediated PDT. (**A**) The induction of ER stress proteins (P-PERK, P-eIF2α, ATF4, CHOP) detected by Western blot analysis. The activity of PERK and eIF2α was determined by phospho-specific antibodies (p-) and then the membranes were reprobed with antibodies to total PERK and eIF2α. Equal protein loading is demonstrated by actin reprobing. As a positive control were used cells treated with 1 µM thapsigargin (TG) for 6 h. (**B**) qRT-PCR analysis of total mRNA isolated from PDT-treated 4T1 and HL60 cells. cDNA was prepared from total mRNA and quantitative real-time RT-PCR for ATF4, CHOP and β-actin mRNA was performed. mRNA fold induction values were calculated from ATF4/actin and CHOP/actin ratios as described in Materials and Methods. Experiments were performed in triplicate.

Any insult that disturbs ER homeostasis ultimately results in ER stress due to the accumulation of misfolded proteins (UPR response) [9]. To analyze whether EG-porphyrins trigger UPR, we first assayed stress sensor PERK. The PERK pathway is activated through phosphorylation of PERK and phosphorylation of its substrate eIF2α. Indeed, immediately after irradiation (time 0) of mTPP(EG)4-loaded cells an evident increase of PERK phosphorylation and phosphorylation of its substrate eIF2α was observed. No such response was detected using para derivatives pTPP(EG)4 and pTPPF(EG)4 (Fig. 8A). Accordingly, the expression of ATF4 and CHOP/GADD153 transcription factors was dramatically increased following irradiation (time 0 and 0.5, respectively). A similar pattern of the PERK pathway activation was detected in human HL60 cells (Fig. 8A, lower panel). ATF4 and CHOP mRNA expression was markedly increased by mTPP(EG)4-PDT, but only modestly by pTPP(EG)4-PDT in both cell types (Fig. 8B). This is in agreement with the results obtained at the protein level. The role of ER stress in mTPP(EG)4-mediated phototoxicity was further accentuated by experiments using salubrinal. Salubrinal inhibits eIF2α dephosphorylation and is known to protect cells from ER stress by reducing translational activity and lowering protein load [28]. Accordingly, salubrinal significantly protected cells against mTPP(EG)4-mediated apoptosis (Fig. S2).

In summary, our results indicate that the PERK signaling pathway plays a crucial role in the mTPP(EG)4-mediated apoptosis.

### PERK knockdown and deficiency partly protects against mTPP(EG)4-mediated apoptosis

**Figure 9:**
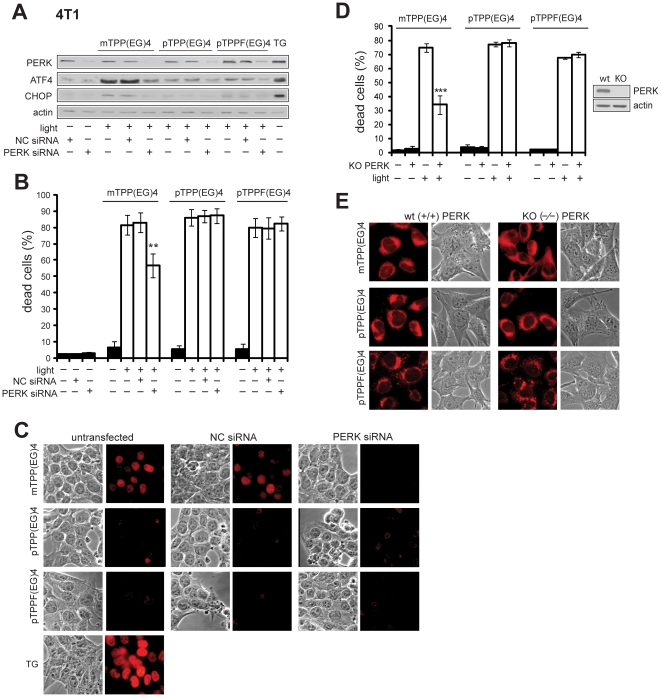
Effect of PERK siRNA on the demise of PDT-treated cells. PERK deficiency protects against mPTT(EG)4-mediated cell death. (**A**) Western blot analysis of PERK, ATF4 and CHOP in 4T1 cells after PERK siRNA transfection. Transfected cells were subjected to EG-porphyrin-mediated PDT, harvested and resolved by Western blot analysis. Equal protein loading is demonstrated by actin reprobing. As a positive control were used cells treated with 1 µM thapsigargin (TG) for 6 h. (**B**) Viability of cells transfected with PERK siRNA. Transfected and control cells were subjected to EG-porphyrin-mediated PDT and cell viability was monitored 24 h later. The percentage of dead cells was expressed as the mean ± SD (n = 7–14). **P<0.01 represents the statistical difference between transfected PDT-treated cells vs. non-transfected or control siRNA-transfected PDT-treated cells. (**C**) Immunostaining of CHOP after EG-porphyrin derivative-mediated PDT in PERK knockdown cells. Cells were fixed 2 h post PDT, immunostained and observed with a fluorescence microscope. The left panel includes untransfected cells, the middle panel cells transfected with control siRNA and the right panel cells transfected with PERK siRNA. (**D**) Cell viability of MEF-wt and MEF-KO evaluated 24 h post-EG-porphyrin-PDT (350 nM mTPP(EG)4, 3.5 µM pTPP(EG)4, 700 nM pTPPF(EG)4). Verification of the PERK−/− phenotype in MEFs-KO cells by Western blot. The percentage of apoptotic cells was expressed as the mean ± SD (n = 6). ***P<0.001 represents statistical differences between PDT-treated MEF-wt cells vs. PDT-treated MEF-KO cells. (**E**) Fluorescence microscopy demonstrates similar cellular uptake of EG-porphyrin derivatives in MEFs-wt and MEF-KO [800 nM mTPP(EG)4, 5 µM pTPP(EG)4, and 2 µM pTPPF(EG)4].

To elucidate the role of the PERK pathway in ER stress evoked by EG-porphyrin PDT, we first knocked down the expression of PERK using specific siRNA. Transfection of PERK siRNA, but not control siRNA (NC), significantly reduced the amount of endogenous PERK protein (Fig. 9A). Moreover, knockdown of PERK abolished induction of ATF4 and CHOP following mTPP(EG)4-PDT (Fig. 9A). A consistent result was obtained using *in situ* immunostaining of CHOP (Fig. 9C). Next we tested the influence of PERK silencing on the viability of 4T1 cells exposed to EG-porphyrin-mediated PDT. Fig. 9B shows that PERK knockdown attenuated cell death induced by mTPP(EG)4, whereas it had no impact on the outcome of para derivatives (pTPP(EG)4 and pTPPF(EG)4) (Fig. 9B).

Secondly, for PDT experiments we used MEFs with the intact (wt) and inactivated PERK −/− (KO) gene. The loss of PERK was verified by Western blot analysis (Fig. 9D). Although the intracellular uptake of EG-porphyrin derivatives determined by porphyrin fluorescence was similar in both cell lines (Fig. 9E), MEFs-KO were remarkably resistant to mTPP(EG)4-PDT. In contrast, no obvious decrease of cell mortality was observed employing para derivatives. This indicates that PERK activation is not required for cell death mediated by para derivatives (Fig. 9D).

In summary, both lines of evidences consistently demonstrate the significance of the PERK pathway in mTPP(EG)4-mediated cell death.

## Discussion

The aim of this work was to dissect the molecular events associated with apoptotic cell death induced by photodynamic activation of several structurally very similar porphyrin derivatives. Extensive research over the past years has demonstrated that photosensitizers used in PDT vary widely in their activity depending on their chemical structure. Differences were even found within a series of molecules with similar basic structural and physicochemical characteristics. In our previous study, we reported that porphyrin derivative substituted with EG chains in para phenyl position exhibited lower biological efficacy (11–13 fold) than its corresponding fluorinated analog [22]. The enhancement of the biological activity by fluorination indicates better molecular targeting due to the presence of fluorine atoms on the phenyl ring leading to altered affinities for specific or non-specific binding sites [22]. Interestingly, in spite of the differences in biological activity, both derivatives target the same organelle (lysosomes) (Fig. 1) and their photoactivation results in the activation of the p38 MAP kinase cascade which is crucial for caspase-dependent mitochondrially driven apoptosis [Fig. 2; [23]].

In contrast, a shift of a glycol chain to the meta-phenyl position resulted in a different intracellular localization. It is known that subcellular localization of the drugs is one of the crucial parameters influencing the efficiency and extent of cell damage, as well as cell response and the mechanism of cell death. Accordingly, this study brings evidence that photoexcitation of mTPP(EG)4 triggers proapoptotic signaling involving different pathways than para derivatives. Nevertheless, some similarities such as immediate p38 and JNK MAPK activation were found. However, the inhibition of p38 activation via pharmacological inhibitors or gene deletion did not interfere with the cell death in contrast to para derivatives. This indicates that p38 kinase does not play a significant role in mTPP(EG)4-mediated apoptosis (Fig. 2). In other studies using various photosensitizers, the role of p38 activation in PDT response appears to be ambiguous [29], [30], [31], [32], [33], [34]. Recently, p38 activation as a death signal has been described for the endocytically located photosensitizer TPPS2a (meso-tetraphenyl-porphine), which is applicable to photochemical internalization, a method used for release of endosomally/lysosomally trapped macromolecular drugs into the cell cytosol to enhance their biological effect in vitro [35]. We can relate our para EG-porphyrin results with these data because they exhibit similar intracellular localization and mechanism involving p38 MAPK activation in the cell death induction. Even though our results seem to further support the concept that the activation patterns and functions of MAPK after photochemical treatment are strongly dependent on the photosensitizer, targeted cell type, cellular localization and PDT regimes, certain common features seem to emerge. However, better understanding of these mechanisms in various paradigms might come from the identification of molecular targets, which are still missing.

The EG chain bound to the phenyl ring in meta position targeted the porphyrin molecule to ER. This localization predisposes cells exposed to PDT treatment to ER stress. In general, oxidative damage to the ER following PDT can result in dramatic changes in ER homeostasis, which can be further propagated to the mitochondrial cell death machinery. ER participates in the initiation of apoptosis by two interconnected mechanisms; unfolded protein response (UPR) and Ca^2+^ signaling [36]. Different ER-accumulated photosensitizers have been shown to stimulate a rapid increase in the intracellular calcium concentrations [Ca^2+^]cyt immediately after photoactivation. Indeed, in our settings we were able to measure a robust Ca^2+^ rise by flow cytometry in both studied cell lines loaded with mTPP(EG)4 (Fig. 4A). A lower rise was observed in the context of para derivatives. Pre-treatment with cell-permeable Ca^2+^ chelator BAPTA-AM completely abrogated this rise (Fig. 4B); however, the same treatment had a different impact on the cell death induced by meta and para derivatives (Fig. 4C). While a substantial increase of cell viability was observed in meta derivative-loaded cells, no protective effect was exerted by BAPTA on the para derivatives. This supports the notion that intracellular Ca^2+^ does not contribute to cell death induced by para derivatives. Moreover, the participation of extracellular Ca^2+^ influxes was excluded by experiments performed in Ca^2+^ free medium or in the presence of extracellular chelator EDTA or EGTA. It should be pointed out that PDT activate multiple pathways and even though para derivatives induce a certain level of ER stress manifested by a moderate Ca^2+^ elevation in the cytoplasm, this likely plays a marginal role in the final cell death. Instead, other mechanisms such as p38 MAPK activation initiate cell death signaling independently of the Ca^2+^ efflux. This notion of multiple pathway activation and participation is supported by the results showing that the protective effect of BAPTA, calpain inhibitor and caspase inhibitors or p38 kinase inhibitor is not complete in the context of either meta or para derivatives (Figs. 2,4,6). One can speculate that these different pathways are in delicate balance and the levels of their activation, including timing, might determine which pathway(s) will dominate and be causative for the development of apoptosis. The remaining question, however, is why the evident strong p38 MAPK activation in mTPP(EG)4-mediated PDT does not play a role in cell death like in para derivatives. The answer might reside in the intracellular location of ROS production and/or the nature of their prevailing species. The application of various ROS scavengers suggested that the major species generated by photoactivation of EG-porphyrins is singlet oxygen (Fig. 3). However, participation of other species depending on the used derivative was indicated by the example of significant formation of hydroxyl radicals in pTPP(EG)4-mediated PDT. The site of ROS generation, including their half life, might also have a strong impact on the molecular targets and activation of signaling cascades. The APF fluorescent probe revealed compartments overlapping with porphyrin localization as primary sites of ROS induction, that is ER for mTPP(EG)4 and lysosomes for p(TPP(EG)4 and pTPPF(EG)4. We must take into account that the demonstrated localization of porphyrin derivatives is not absolute and is also dependent on the used concentration, thus affecting ROS site production.

A precise dissection of individual ROS species and their involvement in triggering specific signals remains to be defined. Further investigation is needed to specify the role of ROS generation sites and re-localization of longer-lasting species such as hydrogen peroxide, whose involvement might be indicated by the moderate inhibitory effect of PEG-catalase on cell death induced by the para derivatives. These questions still remain not fully answered due to the limitation of recent techniques and lack of more specific probes usable for in situ detection.

Importantly, our data showing suppression of [Ca^2+^]cyt by L-histidine pre-treatment correlated with the inhibition of mTPP(EG)4-mediated cell death (Fig. 4). This delineates the order of events and proves ROS as an important initiator of ER stress and Ca^2+^ release.

The inhibition of mitochondrial Ca^2+^ uptake by Ru360 corresponded with the reduction of the cytoplasmic cytochrome c level and significant attenuation of apoptosis (Fig. 5). It substantiates a causative role of intracellular Ca^2+^ rise in mTPP(EG)4-mediated apoptosis, similarly as described in some models [37], [38], [39], [40], [41], [42], [43], [44], and supports the notion that in response to ER stress, the ER–mitochondria cross-talk is facilitated via the release of Ca^2+^ ions from the ER into the cytosol. A close physical contact of mitochondria and ER enables Ca^2+^ fluxes between these organelles and determines the mitochondrial Ca^2+^ responses [45]. When mitochondrial Ca^2+^ uptake is inhibited, the propagation of Ca^2+^ signal into the mitochondria and translation into the mitochondrial cell death machinery is abrogated.

In other models demonstrating the [Ca^2+^]cyt increase, however, different functional outcomes were reported, as having no role [16], [18] or even a protective role [46], [47]. Such discrepancies can be related to the source mediating the rise of the [Ca^2+^]cyt (both influx of extracellular Ca^2+^ and release of Ca^2+^ from different intracellular stores, e.g. ER, mitochondria) and to the specificity of the sensitizers, and particularly of their targets within the cells (reviewed in [3], [48]). In some PDT cases, the increase in [Ca^2+^]cyt was associated with immediate ^1^O_2_-mediated damage to the sarco/endoplasmic reticulum Ca^2+^-ATPase-2 (SERCA2) pump. The resulting incapability of the cells to refill ER Ca^2+^ pools, rather than the increase in [Ca2^+^]cyt per se, were functionally linked to photokilling [18], [49]. Our attempts to define the role of SERCA2, however, did not prove SERCA2 to be a molecular target in mTPP(EG)4-mediated PDT. In contrast to the above-mentioned paradigms, in EG porphyrin-treated cells the SERCA2 protein level diminished with slower kinetics, i.e.1–2 h post irradiation (data not shown) regardless of the sensitizer (similar for both meta and para derivatives). Therefore, the SERCA2 drop in our model rather reflects general protein degradation occurring during the apoptotic process.

The signaling mechanisms transducing the primary photodamage to the ER into cell death pathways in PDT-treated cells have not been completely solved, but a role for the activation of calpains, Ca^2+^-dependent cysteine proteases, has been proposed in recent studies. Calpains were found to be activated either as an alternative mechanism of caspase-independent apoptosis [50] or in parallel to the canonical caspase-dependent pathways [51], [52], [53]. In various ER stress models calpain inhibition led to the reduction of apoptosis [54], [55], [56]. However, an engagement of calpains in PDT models is questionable [51], [57]. The conflicting data can be explained by the differences between the models employed. In addition, some studies suggest that the involvement of calpain in apoptosis seems to be more prominent in certain cell types [58]. In our model, calpain activity demonstrated by fodrin cleavage was significant in mTPP(EG)4-treated cells. It was inhibited by calpain inhibitor PD150606 and ROS scavengers (L-histidine and trolox), but only partly attenuated by BAPTA (Fig. 6). Therefore, it is possible that a portion of the calpain activity might not be Ca^2+^-dependent. Importantly, calpain inhibition correlated with suppression of mTPP(EG)4-mediated cell death in both HL60 and 4T1 cells lines. However, neither BAPTA nor the calpain inhibitor protected cells from the death completely. This strongly indicates that Ca^2+^, besides activating calpain and the Ca^2+^ uptake by mitochondria, has other pro-death roles. A rather unclear role of calpain activation is emerging in the context of para derivatives. The lately discovered cross-talk between the caspase and calpain proteolytic systems brings another level of complexity [59], [60]. It was shown that caspases, including caspase-3, can promote calpain activity through cleavage of calpastatin, the endogenous protein inhibitor of calpains [61], and thus regulate calpain activity during apoptotic cell death. In our setting the calpain activity monitored by fodrin cleavage exhibited slower kinetics with para derivatives than with mTPP(EG)4 (1–2 h versus 0.5 h post PDT) (Fig. 6A) and occurred simultaneously with caspase activation. This could indicate that most of calpain activity in para derivatives is not dependent on Ca^2+^ but rather results from caspase activation. In favor of this interpretation is the insensitivity to BAPTA treatment (Fig. 6B) and reduction of so-called “calpain specific” 150 kDa fodrin fragment by broad-spectrum caspase inhibitor Z-VAD-FMK (Fig. 6D). In addition, both the calpain inhibitor and BAPTA did not show any impact on cell death in the context of para derivatives. Similar results were obtained with bisulfonated aluminum phthalocyanine (AlPcS2) in lymphoblastoid CCRF-CEM cells [51], which together with pTPP(EG)4 and pTPPF(EG)4 represent photosensitizers without significant role of calpains in PDT.

In agreement with data obtained using the inhibitors, knockdown of calpain with specific Capn4 siRNA brought another line of evidence confirming significant reduction of mTPP(EG)4-mediated cell death in 4T1 cells (Fig. 7) but no effect in combination with para porphyrins (Figs. 6, 7). Taken together, activation of calpains is an essential step in the mTPP(EG)4- but not in the pTPP(EG)4-and pTPPF(EG)4-mediated apoptosis. Preliminary experiments implementing capn4 KO MEFs support such conclusion.

In rodent cells, prolonged ER stress stimulates the activation of pro-caspase-12 [6]. This ER membrane-localized enzyme is cleaved and activated by calpain during ER stress, or in response to the mobilization of intracellular Ca^2+^ stores [62]. In mTPP(EG)4-treated cells the activation of caspase-12 and human caspase-4 appeared simultaneously with the cleavage of fodrin, which is a substrate of calpain action [27]. However, we were not able to effectively block caspase-12 and -4 activation by calpain inhibitor to confirm a direct link between calpain and caspase activity (preliminary data). There are at least two possible explanations: either the activation of stress-related caspases is not mediated by calpains but by some other mechanism in our model, or the calpain inhibitor affects some caspase-independent pathway(s) participating in mTPP(EG)4-mediated cell death. A novel function of Ca^2+^ calpain in the processing of mitochondrial apoptosis-inducing factor (AIF), which mediates caspase-independent apoptotic cell death, has been reported recently [63] and seems to be an appropriate candidate to explore in the context of mTPP(EG)4-mediated apoptosis.

In addition, our study shows that unfolded protein response (UPR) participates in the initiation of apoptosis mediated by mTPP(EG)4-PDT. The PERK pathway is activated through phosphorylation of PERK and phosphorylation of its substrate eIF2α immediately after irradiation (Fig. 8A). Subsequently, the full induction of transcription factors ATF4 and CHOP (Fig. 8A, 8B), which are relevant for the regulation of stress response genes, occurs. It has been reported that under conditions of persistent ER stress CHOP induction is an important element of the switch from pro-survival to pro-death signaling [9]. Moreover, PDT-mediated apoptosis with a mitochondrial and ER-localizing porphyrin was reduced in chop-deficient cells, thereby confirming CHOP contribution to the induction of apoptosis in photosensitized cells [64]. In experiments using cells transfected with PERK siRNA, no detectable CHOP expression was detected in contrast to control siRNA after PDT (Fig. 9A). In addition, PERK knockdown and PERK deficiency (Fig. 9) protected cells against mTPP(EG)4-mediated apoptosis. Taken together, our study clearly shows that the PERK pathway activation is a crucial triggering point of ER stress evoked by photodynamic therapy. Although several previous reports based on expression profiling indicated activation of the PERK-eIF2a-ATF4 branch of UPR [65], [66] and were validated at the protein level [67], [68], to our best knowledge our study brings the first evidence that the PERK pathway actually plays a causative role in PDT-induced apoptosis. Interestingly, we were not able to detect activation of other two UPR sensors, IRE1 and ATF6 (data not shown), which were described in other ER stress models [reviewed by [9]]. So far, only two reports have described the possible involvement of IRE1-XBP1 in PDT [65], [67] but no report demonstrated ATF6 engagement. It could indicate that activation of this UPR sensor is not of use in the PDT context.

Based on our current knowledge, we hypothesize that photoactivation of EG-porphyrin derivatives results in the formation of ^1^O_2_ and other ROS species, which as primary messengers trigger multiple signaling pathways leading to cell death. In dependence on the intracellular localization and molecular target(s), various sets of causative and additive pathways are activated. Photostimulation of lysosomally accumulated para EG-porphyrin derivatives thus leads to activation of p38 MAPK, which in turn induces the mitochondrial pathway. Another signal might be sent out by proteases released from lysosomes that in turn activate caspases and Bid processing, thereby promoting the apoptotic process [23]. In contrast, photostimulation of the meta porphyrin derivative accumulated in ER induces dramatic changes in ER homeostasis and activates the Ca^2+^ signaling pathway and UPR via the PERK pathway. Although identification of all the steps involving signal transduction has not yet been achieved, cell death signals can be further propagated into mitochondrial cell death machinery and converged into the caspase-dependent and/or caspase-independent cascade. The apoptotic outcome includes the contribution of additive secondary signals as well. Although para-EG porphyrin derivatives did not seem to primarily induce Ca^2+^ release and PERK pathway activation, these events seems to take place in later stages when compartmentalization of the photosensitizer is changed due to the damage to cellular structures and/or their permeability.

## Materials and Methods

### Photosensitizers

Synthesis and elemental analysis of porphyrin derivatives with the ethylene glycol (EG) chain at meta (mTPP(EG)4 = compound **2**) or para [pTPP(EG)4 = compound **4**, pTPPF(EG)4 = compound **6**] positions were performed at the Institute of Chemical Technology (Prague) and were described elsewhere [22].

### Antibodies

Antibodies recognizing phospho-PERK (Thr980), phospho-eIF2α (Ser51), phospho-c-Jun (Ser73), phospho-p38 MAPK (Thr180/Tyr182), PERK, eIF2α, p38 MAPK, CHOP, caspase-12, caspase-3, caspase-9 were from Cell Signaling Technology Inc. (Beverly, MA, USA), ATF4 from ProteinTech Group Inc. (Chicago, IL, USA), caspase-4 from MBL International Corporation (Woburn, MA, USA), fodrin from Enzo Life Sciences (Farmingdale, NY, USA), CREB-2 (ATF4), c-Jun from Santa Cruz Biotechnology (Santa Cruz, CA, USA), cytochrome c, PARP from BD Bioscience Pharmingen (San Diego, CA, USA) and actin from Sigma Aldrich (St Louis, MO, USA). GAPDH antibody was from GeneTex (Irvine, CA, USA). Antibody against m-calpain was kindly provided by Dr. Peter Greer and used as described previously [69]. Secondary anti-rabbit and anti-mouse antibodies conjugated with horse radish peroxidase, fluorescence secondary antibody Cy3 anti mouse were purchased from Jackson ImmunoResearch Laboratories (Bar Harbor, ME, USA).

### Inhibitors

Kinase inhibitors JNK II (SP600125) and p38 (PD 169316) were purchased from Calbiochem (San Diego, CA, USA), caspase inhibitor Z-VAD-FMK was from Alexis (Lausen, Switzerland), ROS scavengers L-histidine, trolox (6-Hydroxy-2,5,7,8-tetramethylchromane-2-carboxylic acid), NAC (N-Acetyl-L-cysteine), tiron (4,5-dihydroxy-1,3-benzene disulfonic acid), DMSO (dimethyl sulfoxide), PEG-catalase, ionomycin and thapsigargin from Sigma Aldrich (St Louis, MO, USA), PD150606, Ru360 and inhibitor of eIF2α dephosphorylation (salubrinal) from Calbiochem (San Diego, CA, USA). Calcium chelator BAPTA-AM (1,2-bis-(o-Aminophenoxy)ethane-N,N,N′,N′-tetraacetic Acid Tetra-(acetoxymethyl)ester) and sodium pyruvate were obtained from Invitrogen (Carlsbad, CA, USA).

### Cell cultures

All cell lines were purchased from the American Type Culture Collection (Manassas, VA, USA). Cell lines HL60 (human promyelotic leukemia), 4T1 (mouse mammary carcinoma) were kept at exponential growth in RPMI 1640 medium supplemented with 10% fetal calf serum. MEFs-wild-type (wt) and p38α−/− (KO), were kindly provided by Dr. Angel Nebreda and MEFs-wild-type (wt) and PERK −/− (KO), a kind gift of Prof. David Ron, were grown in Dulbecco's modified Eagle's medium supplemented with antibiotics, 10% fetal calf serum, β-mercaptoethanol (55 mM final concentration) and nonessential amino acids.

### Photosensitization

HL60 (human promyelotic leukemia), 4T1 (mouse mammary carcinoma) cells were cultivated overnight at 37°C and 5% CO_2_ in the presence of EG-porphyrin derivatives at a concentration of 0.2–3.2 µM to evoke 60–80% PDT mortality. Then the cells were washed, illuminated with filtered light (500–520 nm, 2.5 J/cm^2^) and harvested at various time points after irradiation for further analysis. Cell viability was determined 24 h post-PDT by the trypan blue exclusion method. Control dark experiments (without illumination) were performed in parallel.

### Inhibitor and scavenger experiments

Cells loaded with EG-porphyrin derivatives were washed and preincubated for 1 h prior to light exposure with 15–20 µM p38 MAP kinase inhibitor (PD 169316), 5 µM JNK inhibitor II (SP600125), 20 mM ^1^O_2_ scavenger L-histidine, 4–5 mM ROS scavenger trolox, 10 mM tiron, 5–15 mM NAC, 1% DMSO, 10 mM sodium pyruvate (Na pyruv), 300 U/ml PEG-catalase, 4–5 µM mitochondrial uniporter inhibitor Ru360 or 20 µM calpain inhibitor PD150606. The preincubation in experiments using calcium chelator 5–10 µM BAPTA-AM, 100 µM caspase inhibitor Z-VAD-FMK and specific inhibitor of eIF2α phosphatase enzymes salubrinal (20 µM) lasted 2 hours. After irradiation the cells were further incubated up to next day for viability determination by the trypan blue exclusion method.

### Western immunoblot analysis

4T1 and HL60 cells were lysed at various intervals after EG-porphyrin derivative-mediated PDT and protein was analyzed by immunoblotting as described [70]. Equal protein loading and transfer was verified by Ponceau-S staining of the membrane and actin reprobing. Cytosolic, mitochondrial and nuclear fractions were prepared using a cell fractionation kit (Abcam Ltd., UK) according to the manufacturer's instructions. As internal loading controls for fractionation experiments, we used cytosolic GAPDH and nuclear PARP.

### RT-PCR analysis

RNA was extracted by immediately dissolving the cell pellet in 1 ml Trizol reagent (Invitrogen, Carlsbad, CA, USA) following manufacturer's instructions. Reverse transcription was performed using M-MLV Reverse Transcriptase (Promega, Madison, WI, USA). PCR was performed with GoTaq polymerase (Promega, Madison, WI, USA) and the resulting products were resolved in 1.5% agarose gel. For qPCR, each sample was prepared in triplicate by placing 1 µl of the cDNA template with FastStart SYBR Green Master (Roche Diagnostics, Mannheim, Germany) to a final volume of 10 µl. Samples were amplified by the Light Cycler 480 (Roche Diagnostics, Mannheim, Germany). Primers for 4T1 cells: ATF4 5′-ACCAGTCGGGTTTGGGGGCT-3′ (forward), 5′-TTCCGAGGAGCCCGCCTTGT-3′ (reverse), CHOP 5′-CTGCCTTTCACCTTGGAGAC-3′ (forward), 5′-CGTTTCCTGGGGATGAGATA-3′ (reverse), β-actin 5′-GATCTGGCACCACACCTTCT-3′ (forward), 5′-GGGGTGTTGAAGGTCTCAAA-3′ (reverse). Primers for HL60 cells: ATF4 5′-CCACTAGGTACCGCCAGAAG-3′ (forward), 5′-GCCTTGCGGACCTCTTCTAT-3′ (reverse), CHOP 5′-TCGCCGAGCTCTGATTGACC-3′ (forward), 5′-AAGCCTTCCCCCTGCGTATGT-3′ (reverse), β-actin 5′-GAGTCCTGTGGCATCCACGA-3′ (forward), 5′-AGGAGGAGCAATGATCTTGAT -3′ (reverse). The relative expression levels of each target gene were normalized to the mRNA of the internal standard gene β-actin.

### Transfection

4T1 cells were seeded onto 35 mm dishes and allowed to reach 50% confluence on the day of transfection. The small interfering RNA (siRNA) constructs, the ON-TARGETplus SMART pool PERK (L-044901-00-0010), ON-TARGETplus SMART pool CAPNS1 (L-048840-01-0010) and the non-targeting siRNA control ON-TARGETplus Non-targeting Pool (D-001810-10-05), were used (Dharmacon, Lafayette, CO, USA). Cells were transfected with 50 nM siRNA diluted in RPMI medium using Lipofectamine RNAiMAX reagent (Invitrogen, Carlsbad, CA, USA) according to the manufacturer's transfection protocol. For further analysis the cells were used on third day after the transfection.

### Flow cytometry analysis

Cells pre-loaded overnight with EG-porphyrin derivatives were washed and then stained with 4 µM Fluo-4-AM from Molecular Probes (Invitrogen, Carlsbad, CA, USA) for 30 min. During inflow, HL60 cells were exposed for 30 s and 4T1 cells for 1 min to a laser beam (424 nm) and then Ca^2+^ efflux was detected by a BD LSRII flow cytometer. Next cells were measured using a modified protocol: preincubation for 1 h with ROS scavenger L-histidine (20 mM) or 2 h with calcium chelator BAPTA-AM (5 µM) and continued as described above.

### Confocal microscopy – subcellular localization

The experiments for determination of subcellular localization of EG-porphyrin derivatives in 4T1 cells were performed as described before using co-staining with 500 nM LysoTracker Green, or 250 nM ER-Tracker Blue-White (Molecular Probes) [22]. Cells were examined under a DMI 6000 inverted Leica TCS A0BS SP5 tandem scanning confocal microscope with an Ar (488 nm) laser and a ×100 oil immersion objective.

### ROS detection

For ROS detection 4T1 cells were seeded on coverslips or on Petri dishes and then incubated with EG-porphyrin derivatives in complete medium for 16 h. After incubation, cells were washed and loaded with 10 µM APF (3′-(p-aminophenyl) fluorescein) from Sigma Aldrich (St Louis, MO, USA) present in phenol red-free culture media for 30 min. Dye-loaded cells were stimulated for 10 s under a DM IRB microscope (Leica) by UV light using Leica filter cube A (excitation filter BP 340–380 nm and long pass filter LP 425 nm for emission) and fluorescence images were acquired by the DFC 480 camera using a ×10 or ×63 oil immersion objective and Leica filter cube N2.1 (excitation filter BP 515–560 nm and long pass filter LP 590 nm for emission) for EG-porphyrin derivatives and cube I3 (excitation filter BP 450–490 nm and long pass filter LP 515 nm for emission) for APF fluorescence.

### Immunofluorescence staining

Cells grown on coverslips in 35 mm Petri dishes were incubated with EG-porphyrin derivatives in complete medium for 16 h. After incubation, cells were washed and illuminated with filtered light (2.5 Jcm^−2^). Two hours later the cells were fixed with 3% paraformaldehyde for 30 min, permeabilized with 0.5% Triton and then blocked with 5% BSA at the room temperature. Subsequently, coverslips were incubated with primary antibody against CHOP (diluted 1∶1000, Cell Signaling Technology Inc., Beverly, MA, USA) at the room temperature for 1 h and with Cy3 anti-mouse secondary antibody for 30 min (Jackson ImmunoResearch Laboratories, Bar Harbor, ME, USA). After being washed, slides were mounted in 50% glycerol and staining was visualized under a fluorescence microscope using Leica cube N 2.1 All images were taken under the same settings.

### Porphyrin fluorescence

MEF cells grown on coverslips were incubated with EG-porphyrin derivatives (800 nM mTPP(EG)4, 5 µM pTPP(EG)4, 2 µM pTPPF(EG)4) in complete medium for 16 h. After washing, porphyrin fluorescence was observed under the microscope.

### Statistical analysis

Results are shown as mean values of at least three independent experiments (n) and standard deviation (±SD) represented by bars. The significance of differences was estimated by ANOVA or by t-test. *P<0.050, **P<0.01, ***P<0.001 represent the level of significance (P<0.050 was considered significant). For all statistical analyses GraphPad Software was used.

## Supporting Information

Figure S1 **Changes in intracellular Ca^2+^ concentration in 4T1 cells in response to ionomycin (ionophore that releases calcium from all cellular calcium stores) or thapsigargin (potent inhibitor of the sarco/endoplasmic reticulum Ca^2+^-ATPase-2 (SERCA2)).** 4T1 cells loaded with Ca^2+^ indicator Fluo-4-AM (4 µM) (Molecular Probes) were exposed to 1 µM ionomycin or 1 µM thapsigargin and followed by flow cytometry to monitor Ca^2+^ levels.
(TIF)

Figure S2 **Salubrinal pretreatment increases cell viability after mTPP(EG)4-mediated PDT.** HL60 and 4T1 cells were pretreated for 2 h with 10 and 20 µM salubrinal, respectively, or with DMSO. The numbers of apoptotic cells were determined by the trypan blue exclusion method 24 h post PDT. The percentage of apoptotic cells was expressed as the mean ± SD (n = 3). *P<0.05, ***P<0.001 represents statistical differences between PDT-treated cells vs. PDT-treated cells in the presence of salubrinal.
(TIF)
