# Supplementary figures and images for: Role of ER Stress Response in Photodynamic Therapy: ROS Generated in Different Subcellular Compartments Trigger Diverse Cell Death Pathways
Source: PLoS One. 2012 Mar 5;7(3):e32972. doi: 10.1371/journal.pone.0032972 (PMC3293927; doi:10.1371/journal.pone.0032972)

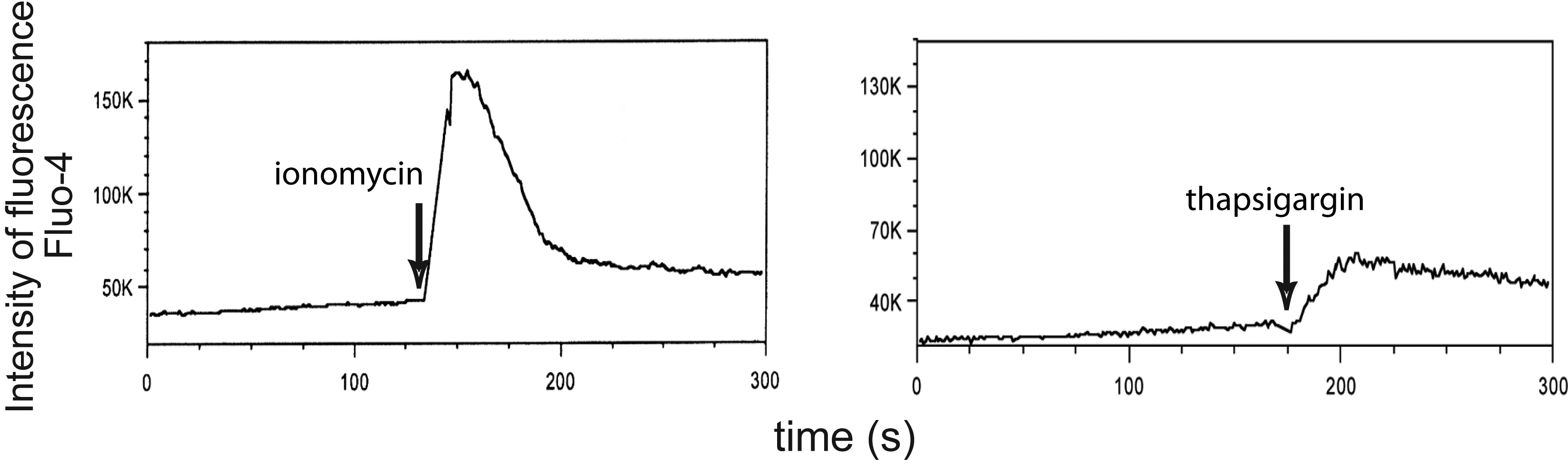

Supplement: Figure S1 — Changes in intracellular Ca2+ concentration in 4T1 cells in response to ionomycin (ionophore that releases calcium from all cellular calcium stores) or thapsigargin (potent inhibitor of the sarco/endoplasmic reticulum Ca2+-ATPase-2 (SERCA2)). 4T1 cells loaded with Ca2+ indicator Fluo-4-AM (4 µM) (Molecular Probes) were exposed to 1 µM ionomycin or 1 µM thapsigargin and followed by flow cytometry to monitor Ca2+ levels. (TIF) [file pone.0032972.s001.tif]

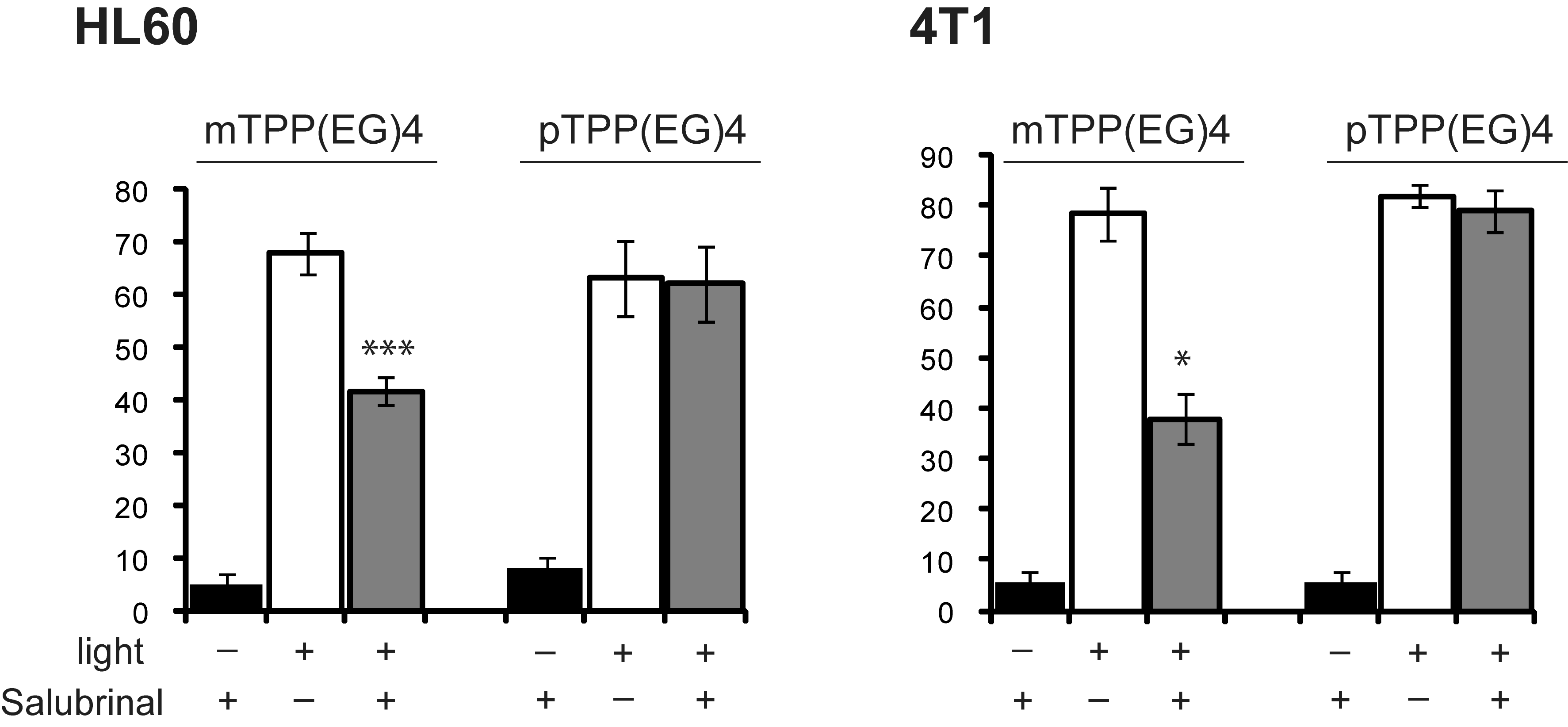

Supplement: Figure S2 — Salubrinal pretreatment increases cell viability after mTPP(EG)4-mediated PDT. HL60 and 4T1 cells were pretreated for 2 h with 10 and 20 µM salubrinal, respectively, or with DMSO. The numbers of apoptotic cells were determined by the trypan blue exclusion method 24 h post PDT. The percentage of apoptotic cells was expressed as the mean ± SD (n = 3). *P<0.05, ***P<0.001 represents statistical differences between PDT-treated cells vs. PDT-treated cells in the presence of salubrinal. (TIF) [file pone.0032972.s002.tif]
